# Effect of *Malva sylvestris* cream on burn injury and wounds in rats

**Published:** 2015

**Authors:** Ebrahim Nasiri, Seyed Jalal Hosseinimehr, Mohammad Azadbakht, Jafar Akbari, Reza Enayati-fard, Sohail Azizi

**Affiliations:** 1 *Traditional and Complementary Medicine Research Center, Faculty of Allied Medical Sciences, Mazandaran University of Medical Sciences, Sari, Iran*; 2 *Traditional and Complementary Medicine Research Center, Faculty of Pharmacy, Mazandaran University of Medical Sciences, Sari, Iran*; 3 *Department of Pharmaceutics, Faculty of Pharmacy, Mazandaran University of Medical Sciences, Sari, Iran.*; 4 *Department of Laboratory Medicine, Faculty of Allied Medical Sciences, Mazandaran University of Medical Sciences, Sari, Iran*

**Keywords:** *Malva sylvestris*, *Burns*, *Silver sulfadiazine*, *Rats*, *Wound healing*

## Abstract

**Objectives:**

Burn injury is one of the most health-threatening problems in the world. *Malva sylvestris (M. sylvestris) *flowers have a high mucilage content and are used as a remedy for cut wound and dermal infected wounds in Iranian folklore Medicine. The purpose of this study was to investigate the effect of *M. sylvestris *cream on the second degree burn injury in rats.

**Materials and Methods:**

Five groups of 10 rats per group were burned with hot metal plate. Animals were administrated divided as control, normal saline, standard silver sulfadiazine 1% (SSD), 5% *M. sylvestris*, and 10% *M. sylvestris* into separate groups. Wound area, percentage of wound contraction, and histological and bacteriological assessments were evaluated.

**Results:**

Wound sizes were not significantly different among groups on 1^st ^and 3^rd^ days after burn injury, while they were significantly different among groups after 7^th^ day post-burn injury. The average areas of wounds on the 15^th^ day were 7.5±2.9, 6.7±2, 10.5±1.6, 4.7±2, and 4.5±2 cm^2^ for base cream, normal saline, SSD, 5% *M. sylvestris,* and 10%* M. sylvestris*, respectively. The results of histology exhibited well-formed horizontally-oriented collagen fibers in MS topical treatment groups. Microorganisms existed in the SSD group were most probably Staphilococcus epidermitis and for NS group were staphylococcus saprophiteccus.

**Conclusion:**

*M. sylvestris* cream improved histological changes of tissue components in the process of healing when compared with SSD cream. Therefore, it can be used as a topical treatment agent for burn wound.

## Introduction

Burn injury is one of the most health-threatening problems in the world (Forjuoh 2006 ▶). Over 6.6 million people world-wide suffer from burns and almost 265000 of them die annually (Penn et al., 2012▶; Mogosanu et al., 2013 ▶). About 1% of all deaths is related to burn injuries (Sadeghi-Bazargani and Mohammadi 2012 ▶). Prevention and management of wound infection are a major factor in wound care. There are many topical agents which are used for burn wound treatment (Khorasani et al., 2009 ▶; Hosseinimehr et al., 2010 ▶). The most important treatment for burn wound is silver sulfadiazine 1% cream (SSD) with antibacterial activity (Miller et al., 2012 ▶ ). SSD may cause side effects such as neutropenia, erythema multiforme, crystalluria, methemoglobinemia (Chung and Herbert 2001 ▶; Fong and Wood 2006 ▶; Beheshti et al., 2013 ▶), and delay wound healing. It is cautioned that SSD cream should not be used for long time on extended wounds (Atiyeh et al., 2007 ▶; Khorasani et al., 2009 ▶; Yaman et al., 2010 ▶). Wound healing process consists of inflammation, re-epithelialization, granulation, neovascularization, and wound contraction. Several natural products have been used for the management of burn wounds that could be considered as an alternative source of treatment of burn wounds. These products have been offered as more effective and cheaper treatment agents (Süntar et al., 2010 ▶; Nasiri et al., 2014 ▶; Bahramsoltani et al., 2014 ▶).

*Malva sylvestris* (Malvaceae), usually known as common mallow, is a native plant to Europe, North Africa, and Asia (Barros et al., 2010▶). In Iran,* M. sylvestris *is known as “Panirak” in folklore. *M*.* sylvestris *has high mucilage content and polysaccharides that are used for many purposes (Aliasl 2013▶; Samavati and Manoochehrizade 2013 ▶; Usami et al., 2013 ▶).This plant has antiulcerogenic activity which is probably related to its high mucilage content. (Samavati and Manoochehrizade 2013 ▶).* M*.* sylvestris *is consumed as a vegetable in Iran. The plant flowers are used as a remedy for cut wound, dermal infected wounds, eczema, and inflammatory disease such as gastritis, bronchitis (Pirbalouti et al., 2009 ▶) (Samavati and Manoochehrizade 2013 ▶), and rheumatism (Conforti et al., 2008 ▶) and is recommended for acne and skin care (Barros, Carvalho et al., 2010 ▶). Other properties of this plant were reported to be diuretic, laxative, spasmolytic, lenitive, choleretic, and antioxidant effects. *M. sylvestris* contains polyphenols, vitamin C, vitamin E, β-carotene (Barros, Carvalho et al., 2010 ▶), anthocyanidines, naphthoquinones, flavonoids or mucilaginous polysaccharides, tetrahydroxylated linear diterpene, monoterpenes, and phenol derivatives (Cutillo et al., 2006 ▶; Veshkorova et al., 2010 ▶; Razavi et al., 2011▶). Dellagreca et al. isolated eleven compounds from aqueous extracts of *M. sylvestris* such as 4-ydroxybenzoic acid, 4-methoxybenzoic acid, ferulic acid, methyl 2-hydroxydihydrocinnamate, and scopoletin as well as malvone A, 2-methyl-3-methoxy-5, and 6-dihydroxy-1,4-naphthoquinone (Pirbalouti et al. 2009▶, Dellagreca et al. 2009 ▶; Pirbalouti, Yousefi et al., 2009 ▶); DellaGreca et al., 2009 ▶). Gasparetto reported additional therapeutic properties related to flowers and aerial parts of *M. sylvestris* such as anti-inflammatory, anticancer, positive effectiveness on gingivitis, abscesses, tooth pain, urological disease, insect bites, and ulcerous wounds (Gasparetto et al., 2012 ▶).

The purpose of this study was to investigate the effect of *M. sylvestris* cream on the second degree burn wounds and compare its results with silver sulfadiazine in rats.

## Material and Methods

**Plant material**

*M* *. sylvestris* flowers were procured from a herbal drug market (Sari, Iran) on September 2013. This herb was confirmed by a senior botanist, Prof. Mohammad Azadbakht, Department of Pharmacognosy, Faculty of pharmacy, Sari, Iran, and a voucher specimen (no. 1002) was deposited at the Department of Pharmacognosy, Mazandaran University of Medical Sciences. The flowers of *M. sylvestris* were dried at room temperature and powdered in a grinder. Aqueous ethanol (70%) was added to the powdered flowers and the mixture was kept at room temperature for 72 hours (Pirbalouti et al. 2009 ▶). After filtration, the solution was concentrated to dryness in reduced pressure under a rotary evaporator. Extract yield was 9.68% w/w. Hydro alcoholic extract of herbal flowers was dried to a powder with the use of a freeze dryer. 

**Formulation of** ***M*** ***. sylvestris*** **cream**

The *M. sylvestris* extract was mixed with liquid paraffin (5 g), stearyl alcohol (5 g), cetyl alcohol (5 g), and span 60 (0.7 g) at 70 ^˚^C. It was prepared by adding tween 80 (1.8 g), propyl (0.015 g), methyl paraben (0.025 g), and glycerin (7g) in distillated 20.5 ml water and was heated to 70^ ˚^C. This formulation was prepared according to result of the previous studies and performed seven times formulation laboratory experience (Pirbalouti et al. 2009 ▶). Then, aqueous phase and oil phase were mixed and homogenized for 15 minutes. The cream was allowed to cool down at room temperature while being homogenized. The concentrations of *M. sylvestris* extract were 10% and 5% in topical creams. All formulations were stored at 4, 25, and 40 ^˚^C for two weeks and then the stability was evaluated. Consistency and uniformity of creams as well as inseparability of the aqueous and oil phase at different times were observed.

**Animal study**

The ethical and research committee of Mazandaran University of Medical Sciences approved the experimental protocol. All animals were obtained from animal house of the Mazandaran University of Medical Sciences. Male rats (n=50) weighing 160-200 grams, average age 10 weeks, were used and housed under standard condition at room temperature with a 12-h light/dark cycle, temperature approximately 22-23 ^˚^C for one week prior to the start of the experiment. Animals were allowed free access to laboratory food and water *ad libitum*. Each rat was weighted and anesthetized by intraperitoneally injection of 50 mg/kg sodium thiopental. Dorsum was shaved using an electric clipper and 70% alcohol was used to disinfect the dorsal area. Deep anesthetized rats were kept in a prone position. A deep second degree burn wound was induced by a hot metallic device (diameter: 5×2.5 cm^2^) warmed for 5 minutes within boiling water and put for 10 seconds on the dorsum of rat skin with an equal weight and pressure (Sayar et al., 2014 ▶; Akhoondinasab et al., 2014 ▶; Haghdoost et al., 2013 ▶). All animals were resuscitated with injection of 5 ml normal saline after burning. The burned animals were randomly divided into five groups of ten rats. Group 1 (NS) was control and rats were only washed with normal saline during dressing without any topical treatment. Group 2 was treated with base cream (BC) without any effective agent. Group 3 was treated with SSD 1% (Behvarzan Pharmaceutical Company, Iran). Animals in groups 4 and 5 were treated with 5% and 10% *M. sylvestris* cream. After topical application of creams, the wound was covered with the sterile plain gauze for 24 hours. The wound area was daily washed with normal saline in all groups, and then was dressed with cream for each group. In order to quantify the rate of wound healing, the wound area was evaluated using a ruler in 1, 3, 7, 10, 15, 20, 25 30, and 35 days after burn injury. The wound area was displayed as cm^2^. The area of wounds at each day was determined by a formula, which represented the area (cm^2^) by length and latitude rectangular. The rectangle area was calculated with length **×** width. Wound contraction was expressed as a reduction in percentage of original wound size. Percentage wound contraction on day X = [(area on day 0 *– *open area on day X) /area on day 0] *× *100 (Tavares Pereira et al., 2012 ▶). Moreover, scales for the burn wound healing were evaluated according to histopathological components in all the groups (Haghdoost et al., 2013▶; Oliveira et al., 2013). 

**Histological study**

Histological examinations of wound repair process were performed in 8 and 21 days after burn injury. After incision, samples were full thickness with 3 mm thickness. The samples were kept in formalin 10%. Tissue incisions were prepared in 5 micron thickness and were stained with hematoxylin-eosin and also specific collagen fibers staining. Masson trichrome stain was used for the examination of density of collagen fibers and blood vessel. Angiogenesis assessment (neovascularization), fibroblastic proliferation and presence of collagen fibers, re-epithelialization, complete healing, and infiltration of inflammatory cell were evaluated in tissue sections. Horizontal section of the microscopic high power field (HPF) was evaluated. Average value of the results of 10 HPF microscopic fields was calculated. The score were calculated as follow:

0 or (-) score was defined as the absence of vessels and cells such as macrophage in each HPF.

Angiogenesis of mild score was defined by the presence of 1 to 2 vessels, and for the cell study, it was defined as the presence of 1-2 cells such as macrophages which scored, 1 or (+).

Moderate score was 2 or (++), which meant the presence of 3-4 vessels for angiogenesis and 3-4 cells for cell study in each HPF. 

Severe condition was 3 score or (+++) which was the presence of 5 or more cells or vessels in HPF.

Polymorphonuclear leukocytes (PNM) were used to assess pathological changes. These tissue sections were assessed by a pathologist blinded to the treatment assignment.

Histological criteria were defined according to a modified scoring system for surgical wound healing taken from previous studies (Velnar and Bailey, 2009 ▶; Tavares Pereira et al., 2012▶; Haghdoost et al., 2013 ▶; Akhoondinasab et al., 2014 ▶). 

The sum of scores for wound healing was divided into three categories: extent of granulation tissue was scored based on seven parameters (the re-epithelization was monitored by evaluation of six components. The sum of scores for re-epithelization had a range from 0 to 18. Eighteen was the highest degree of re-epithelization on the 8^th^ day and the new dermis was evaluated with five components. The sum of scores for granulation state was a range from -3 to 18. Eighteen was the highest degree of granulation tissue formation on the 8 days after burn injury. The sum of the scores for re-epithelization was a range from 0 to 18. 

New dermis was evaluated with five components. The sum of the scores was between 0 and 15. The score for the best condition for new dermis was 15 in 21 days after burn injury. Each part of the categories or each component scored 0-3. The complete healing was evaluated on 21^st^ day after burn injury. The sum of the scores for wound healing was devided into four groups as follow: 0 = no healing, (1-5) = low, (6-10) = moderate healing, and (11-15) = good healing). According to mathematical logic, the sum of the scales for each part of histopathological evaluation was devided into three groups: extent of granulation, new dermis, and re-epithelization. These criteria which were used as histological scores of wound healings are summarized in Table 1. This scoring system was used to determine the healing grade in each treatment group sample (Tavares Pereira et al., 2012 ▶; Edraki et al., 2014 ▶)*.*

**Microbiological** **evaluation**

Swabs were taken from the wounds during dressing change on 4^th^ and 8^th^ days. The collected swabs were immediately transferred to the laboratory for microbial tests. In the quantitative count study, 0.5 ml of normal saline was added to each sample tube. Each sample dilution was spread onto blood agar and MacConkey agar, and the plates were incubated at 37 °C for 24 hours and then the degree of contamination of injuries was evaluate. Diagnostic test for the colonies was applied to Novobiocin test.

**Table 1:** Scales for the burn wound healing according to histopathological components in M.* sylvestris* and control groups of second-degree burn

**Sum of components variables**	**Difinition of Scales**
1- Extent of granulation tissue (7 parameters) [(-3) -18]	(-3-0) = not healing (1-4) = low (5-12) = moderate healing (13-18) = good wound healing
2- re-epithelization ( 6 parameters) [0-18]	0 = not healing (1-6) = low (7-12) = moderate healing (13-18) = good wound healing
3-new dermis (5 parameters) [0-15]	0 = not healing (1-5) = low (6-10) = moderate healing (11-15) = good wound healing
4-Sum of three components	[(-3) - 0] = not healing [1--18) = low (19-37) = moderate healing (38-57) = good wound healing

**Wound observation**

Wound sites were assessed daily. Macroscopic visual evaluation was measured by direct observation of wounds during dressing each day. Tissue inflammation was evaluated by studying edema, secretion, redness, dark secretion or pus and wound bleeding, blistering, swelling, crust, during dressing at which follow-up visit, and wound area was recorded as being absent, redness, edema, or dirty and with light or dark secretion. The wound contraction was also evaluated .

**Statistical analysis**

Statistical analysis was carried out using the SPSS (Version 15) software. The data were tested for normality. One-way analysis of variance (ANOVA) was used for comparing quantity variables in the groups, followed by a post hoc multiple comparing test. Kruskal Wallis H test was used for qualitative variables between groups. The difference data were considered significant at p<0.05.

## Results

The effect of burn injury on losing weight of rats is shown in Table 2. No significant differences were observed between *M. sylvestris* and control groups in the loss of weight after burn injury. Skin wound area was measured at 1, 3, 7, 10, 15, 20, 25, 30, and 35 days after the burn injury.

The average area of wound on the 7^th^ day was 11.4±3.7, 10.6±2.7, 13±1.6, 10±3, and 8.7±1.8 cm^2^ in base cream, NS, SSD, 5% *M. sylvestris,* and 10% *M. sylvestris* creams, respectively (p<0.05). The wound size had normal distribution which was shown using one-sample Kolmogorov-Smirnov test. Wound sizes were not significantly different between groups on 1^st ^and 3^rd^ days after burn injury. Wound area was significantly different between groups on 7^th^ day of the post-burn injury and following multiple comparison Dunnett post hoc test showed that *M. sylvestris* 10% cream group exhibited lower wound size compared to SSD (p<0.05). There were not any significant differences between 5% *M. sylvestris*, 10% *M. sylvestris* and the base cream (Table 3).

**Table 2:** Mean weights (gram) of the rats after burn injury in different groups

**Group**	**1** ^th^ ** day**	**3** ^th^ ** day**	**7** ^th^ ** day**	**15** ^th^ ** day**	**20** ^th^ ** day**	**25** ^th^ ** day**
BC	184±16	182±15	190±14	203±15	215±19	224±20
N/S	183±15	180±16	184±14	206±10	212±13	216±16
SSD	185±12	182±11	185±12	195±12	207±15	216±13
MS5%	186±19	185±16	195±24	201±25	215±18	224±18
MS1%	184±17	183±17	187±19	201±16	214±11	229±9
p value*	0.991	0.956	0.678	0.766	0.801	0.739

BC: Base cream, N/S: Normal saline, SSD: Silver sulfadiazine, MS: *M. sylvestris*

* p value of differences between groups.

**Table 3:** Mean wound area (cm^2^) of the animals groups treated with various topical ointments in post burn injury.

**Group**	**1** ^st^ ** day**	**3** ^rd^ ** day**	**7** ^th^ ** day**	**10** ^th^ ** day**	**15** ^th^ ** day**	**20** ^th^ ** day**	**25** ^th^ ** day**	**30** ^th^ ** day**
BC	12.5±3	13.3±3	11.4±3.7	10.8±3	7.5±2.9	5±1.7	1.4±0.8	0.4±0.5
N/S	11.8±2.4	12.6±2.1	10.6±2.7	9±3.1	6.7±2	4.4±2	1±0.8	0.2±0.3
SSD 1%	13.3±2.5	13.3±2.5	13±1.6	12.6±2	10.5±1.6	5.5±1.5	3.1±1.3	1.8±0.7
MS 5%	12.9±3.2	13.5±3	10±3	9.7±2.6	4.7±2	0.8±0.7	0.41±0.44	0
MS 10%	11.6±2.4	12±1.9	8.7±1.8	8.4±2	4.5±2	0.4±0.5	0	0
*p* value*	0.575	0.675	0.014	0.011	0.001	0.001	0.001	0.001

BC: Base cream, N/S: Normal saline, SSD: Silver sulfadiazine, MS: *Malva sylvestris.*

**Figure 1:**
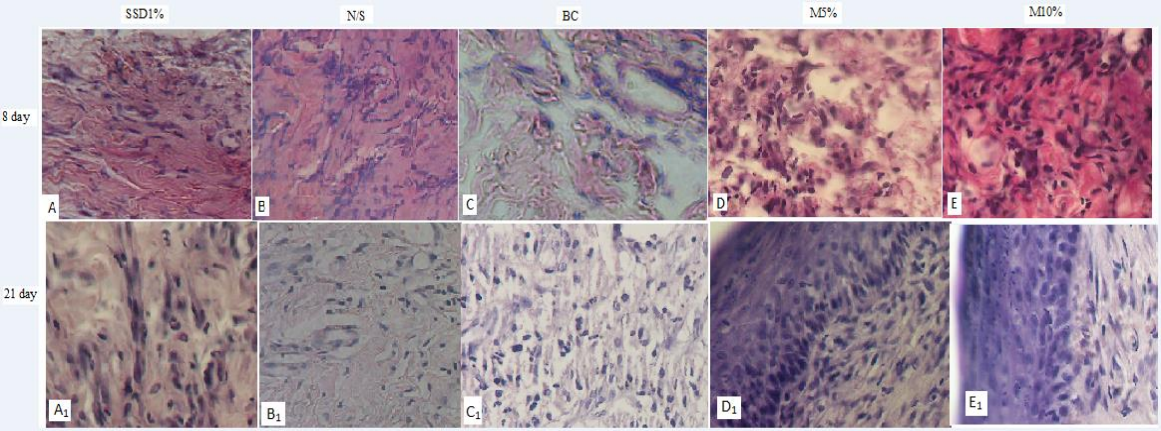
Comparison of histopathology view among *M. sylvestris* (MS) treatment and control groups on 8^th^ and 21st days after burn injury.( A, A_1_ = SSD1% group, B, B_1_= NS, C, C_1_ = BC, D, D_1_ = MS 5%, and E, E_1_= MS 10% group). [basic cream (BC), normal saline (NS), standard silver sulfadiazine treated (SSD 1%),* M. sylvestris* 5 % (MS5%), and *M. sylvestris* 10 % (MS 10%) creams], Macrophage infiltration, neovascularization activity and fibroblastic proliferation were better in *M*. *sylvestris*, compared with control groups on the 8^th^ day. Moreover, degree of scar formation, collagenization organization, and new dermis formation in *M. sylvestris* were better in herbal treatment groups in comparison with control groups on 21st day

In the base cream and SSD groups, skin irritation such as redness, edema, and slight secretion were observed in 5, 8, 12 days post-burn injury. The wound area was not different between 5% and 10% *M. sylvestris* 15 days after burn injury (p<0.32), meanwhile, it was different between SSD and base on day 8 which biopsy samples showed an increased macrophage infiltration and fibroblastic proliferation in animals that were treated with 10% *M. sylvestris*or 5% *M. sylvestris*, which were better than SSD, NS, and base cream groups (Figure 1 and Table 4) (p<0.05). 

**Table 4:** Extent of granulation tissue examined in different groups on 8^th^ day after burn injury

**Components/Groups**	**BC**	**SSD 1%**	**N/S**	**MS 5%**	**MS 10%**
Macrophage histocytic infiltration	2	2	2	3	3
Neovascularization	1	2	2	1	3
Fibroblastic proliferation	2	2	2	3	2
Matrix mucopolisacharid deposition	2	2	2	2	2
Degree of inflammation	3	3	1	3	3
Extent of bacterial colonization	-1	-2	-1	-1	-1
Degree of granulation tissue formation	1	2	1	1	2
Sum	10	11	9	12	14

BC: Base cream, N/S: Normal saline, SSD: Silver sulfadiazine, MS: *M. sylvestris*

**Figure 2:**
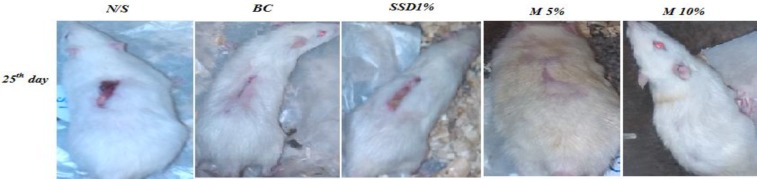
Burn wound healing pattern in control groups [normal saline (NS), base cream (BC), and silver sulfadiazine 1% (SSD1%)] and herbal treated groups [*M. sylvestris* cream 5% and 10% (MS 5%,MS 10%)] in rats. The rate of healing in burn wounds created on rats were measured and photographed at regular intervals in both control groups and herbal treated rats during a 25-day period. Wound healing condition in (MS 10%) and MS 5% from ten rats were completed on 25 days

**Figure 3:**
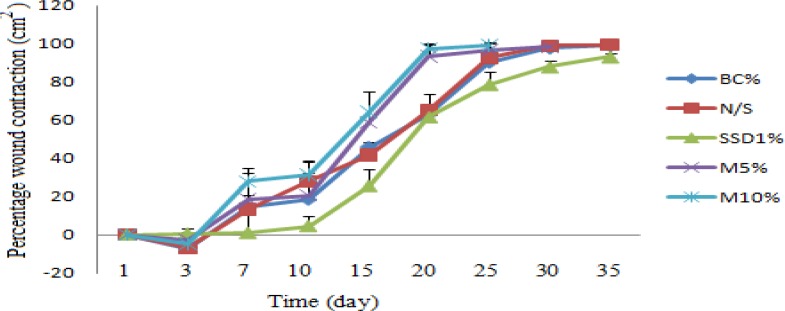
Comparison of the percentage of wound contraction between *M. sylvestris* *and *control groups. *M.sylvestris *(MS) 5% and10% creams treated groupd showed faster time than control groups for wound contraction (BC: Base cream, N/S: Normal saline, SSD: Silver sulfadiazine, MS: *M. sylvestris*

The percentage of wound contractions in different groups is shown in Table 5. It was significantly increased in 10% *M. sylvestris* group as compared to the SSD and control groups. Animals who received herbal treatment creams had a shorter healing time than rats in all control groups (Figure 2). Wound contraction started from day 4 in treatment group and day 5 in control groups. On day 7, animals treated with 10% *M. sylvestris* exhibited significant increase in the percentage of wound contraction as compared to other experimental groups. On day 20 of post-burn injury, 10% and 5% *M. sylvestris* creams exhibited more than 90% wound healing, whereas it was 63% , 65.1%, and 61.5% in rats treated with BC, NS, and SSD creams, respectively. On day 25, no scar was observed in animals treated with 10% and 5% *M. sylvestris*, while this improvement was observed for control groups on day 30. The time of wound healing in the herbal treatment group was about 10 days shorter than the SSD group. The herbal groups were treated 5-7 days faster than the BC group (Table 5 and Figure 3).

**Table 5:** Comparison of the percentage of wound contraction among *M. sylvestris *(MS) and control groups

**Group**	**3** ^rd^ ** day** **(%)**	**7** ^th^ ** day (%)**	**10** ^th^ ** day (%)**	**15** ^th^ ** day (%)**	**20** ^th^ ** day (%)**	**25** ^th^ ** day (%)**	**30** ^th^ ** day (%)**	**35** ^th^ ** day (%)**
BC	-7±6.5	14.6±16	18.4±9.8.	45.9±9.6	63±9.3	90.2±3.5	97.6±33	98.8±1.8
NS	-7±4.2	13.4±7.4	28±11	41.9±6.5	65.1±8.5	92.6±4.1	98.8±1.9	99.2±2(32D)
SSD 1%	.5±3	.9±11.6	4.5±5.2	25.6±8.6	61.6±6.7	78.8±6.6	88±3.4	93.2±2
MS 5%	-4±4.3	18.6±16.3.5	20.6±12	58.9±16.2	93.2±6.1	96.5±.3.4	98.3±2.9	100
MS 10%	-4%±7.9	28±4.6	31.4±6.8	64.4±10.3	97.3±2.9	99±1.6	100	100

BC: Base cream, N/S: Normal saline, SSD: Silver sulfadiazine, MS: *M. sylvestris.*

The histopathology results of the condition of granulation tissue, matrix of organization, re-epithelialization, and new dermis generation are explained in Tables 4, 6, and 7. Histopathological results showed that the highest and the lowest extent of granulation tissue were observed in 10% *M. sylvestris* and NS groups, respectively (Table 4). Tissue re-epithelialization components were improved in herbal groups as compared to SSD and other control groups on 8 days after burn injury. Thickness of the granular cell layer and the maturation organization of squamous cells and orthokeratin in herbal treatment group were improved compared to control group. The sum of the re-epithelialization parameters score of herbal treatment was much more than SSD and N/S treatment group (Table 6 and Figure 1).

Matrix of collagenization organization in 10% *M. sylvestris* group was better than all of the control groups at 21^st^ day. Furthermore, the score of degree of scar formation was better in a herbal treatment group. These details are shown in Table 7. Histopathological results showed that complete wound healing and new dermis formation were observed in 10% and 5% *M. sylvestris* groups whereas SSD, NS, and base cream groups had moderate wound healing. 

**Table 6:** Comparison of the re-epithelialization components between *Mavla sylvestris* (MS) and control groups on 8^th^ day post-burn injury

**Group/component**	**Epidermal** **thickness (0-3)**	**Thickness of granular cell layer**	**Maturation organization of squamous cells**	**Extent of Keratin layer**	**Orthokeratin**	**Parakeratosis**	**Sum**
BC	1	1	0	0	0	0	2
N/S	1	0	0	0	0	0	1
SSD 1%	1	1	1	1	1	1	6
MS 5%	3	3	3	3	3	0	15
MS 10%	3	3	3	3	3	0	15

BC: Base cream, N/S: Normal saline, SSD: Silver sulfadiazine, MS: *M. sylvestris.*

**Table 7:** Comparison of the new dermis formation between *M. sylvestris* (MS) and control groups on 21^st^ day post-burn injury time

**Groups**	**Degree of scar formation**	**Collagenization organization**	**Extent of hair folliculs **	**Extent of lymphatic ducts**	**Degree of innervations**	**SUM**
BC	2	2	0	0	0	4
N/S	2	2	1	0	0	5
SSD 1%	2	2	1	0	0	5
MS 5%	3	2	1	1	1	8
MS10%	3	3	2	1	1	10

BC: Base cream, N/S: Normal saline, SSD: Silver sulfadiazine, MS: *M. sylvestris.*

**Table 8:** The sum of the score of three histopathological components for burn wound healing in *M. sylvestris *(MS) treatment and control groups

**Group**	**Re-epithelialization** **[0-15]**	**Extent of granulation tissue[(-3)-18]**	**New dermis formation[0-15]**	**Sum of score** **[(-3)-58]**
BC	2	10	4	16
NS	1	9	5	15
SSD 1%	6	11	5	22
MS 5%	15	12	8	35
MS 10%	15	14	10	39

The extent of granulation tissue in herbal treatment groups was better than normal saline group. Moreover, parameters of the new dermis in *M. sylvestris* were better than SSD and other control groups. Three main histopathology components of wound healing in all groups are shown in Table 7.

Histopathological data showed that 10% and 5% *M. sylvestris* had better healing effects as compared with control groups (Table 8). 

The period of re-epithelialization among the study groups were different. 

The reepithelialization time for 10% *M. sylvestris* was 25 days after burns injury (99±1.6%). This time for SSD cream was 35 days (93.2±2%). A completed wound healing was observed in animals treated with 10% and 5% *M. sylvestris* on 30^th^ and 35^th^ days, respectively, while this time for SSD, NS, and BC creams were more than 35 days. We observed that wound care in the rats treated with herbal creams was five days shorter than the control groups.

Laboratory evaluation indicated no evidence of pathological bacteria in 4^th^ and 8^th^ days. On day 8, a little colorless secretion was observed on wounds in SSD and NS groups and bacteria were grown on blood agar culture media. Catalase positive gram positive cocci was observed in samples of these groups. The results of bacitrucin sensitive and coagulase tests for these samples were negative. This process was completed by novobiocin test which is used to differentiate coagulase-negative staphylococci. The SSD group sample was sensitive to the novobiocin test; on the contrary, NS group sample was not sensitive to this test. Finally, microorganism existed in the SSD group was most probably Staphilococcus epidermitis while for NS group, it was Staphylococcus saprophiteccus. MacConkey agar culture did not show any pathologic organism growth in these groups.

## Discussion

The purpose of this study was to evaluate the effect *M. sylvestris* topical cream on burn wound healing in rats. The main result of this study showed a significant increase in burn wound contraction with topical 10% and 5% *M. sylvestris* cream during experimental trial, as compared with SSD, BC, and NS groups. The animals treated with the *M. sylvestris* showed a significant reduction in the wound area when compared with other groups. Wound healing in 10% and 5% *M. sylvestris* creams treatment groups was about 90% on the 20^th^ day after burn injury, whereas it was 63%, 65.1%, and 61.5% in rats treated with BC, NS, and SSD creams, respectively. Pathological bacteria did not exist on burn wounds that treated with herbal creams. In previous studies, *M. sylvestris* was used topically for treatment of various diseases such as ulcers, dermatitis, swellings, abscesses, cough, bronchitis, inflammatory diseases, and burns (Chung and Herbert 2001 ▶; Camejo-Rodrigues et al., 2003 ▶; Razavi et al., 2011 ▶; Wang 2005 ▶; Pirbalouti et al., 2009 ▶). Many components of the *M. sylvestris* can be responsible for antimicrobial activity against pathogen microorganisms (Veshkorova et al., 2010 ▶; Razavi et al., 2011 ▶).

The chemical compositions of *M. sylvestris* were reported to be malvone A: 2-methyl-3-methoxy-5, 6-dihydroxy-1,4-naphthoquinone as flavonoids. Excretion of free radicals, antioxidant action, and anti-inflammatory properties of this plant showed to contribute to the treatment of wound (Pirbalouti and Koohpyeh 2011 ▶). Carotenoids, high vitamin C, carbohydrates and particularly sugars such as fructose and glucose and phenolics and high amount of ascorbic acid are present in the flowers of this plant (Barros, Carvalho et al., 2010▶). The results of bacteriology tests on the wound of *M.* *sylvestris* treated animals did not have showed any pathologic bacteria and doubtful secretion. Malvone A in *M. sylvestris* flower extract may be responsible for antibacterial activity (Pirbalouti and Koohpyeh 2011 ▶). Moreover, antioxidant, high vitamin C, and anti- inflammatory activities of *M. sylvestris* could be potent and effective properties of this plant for improving of wound healing and increasing wound contraction. 

Our finding showed that 10% and 5% *M. sylvestris* could effectively prevent swelling, erythema, secretion, and other burn complications. The best results for wound contraction and short time of wound healing were obtained in animal treated by M. *sylvestris* cream. Delaying of the wound healing rate in control groups as compared to topical herbal treatment may be related to existence of bacteria in wounds or because of their histopathological lesions. Although many studies reported that SSD cream is widely used in burns units to reduce the risk of secondary infection and proposed as a standard treatment for burn wound, but we found microorganisms in SSD group. This might be the reason of many problems such as delay in wound repair (Fuller 2009 ▶; Hosseinimehr et al., 2010 ▶; Maghsoudi et al., 2011 ▶). Malvone A in *M. sylvestris* may be responsible for antimicrobial activity. This may be due to either the individual or additive effects of the phyto-constituents that accelerate the process of wound healing (Pirbalouti and Koohpyeh 2011▶). Razavi et al. have reported that the flower extract of *M. sylvestris* showed high antibacterial effects against some human pathogenic bacteria strains. They concluded that it can be considered as an antiseptic agent (Razavi et al., 2011 ▶). Antibacterial properties of *M. sylvestris* can help to prevent the wound infection. In recent experimental studies, application of the *M. sylvestris* cream on the wound was associated with significant wound healing (Razavi et al., 2011▶; Pirbalouti et al., 2009 ▶). 

In histopathological assessment, the re-epithelialization was also found to be significantly better in animals treated with creams containing *M. sylvestris*. The results of histological evaluation showed that *M. sylvestris* significantly increased the rate of collagen turnover and wound reduction. Previous studies showed that *M. sylvestris* cream could increase well-organized bands of collagen and the number of fibroblasts and few inflammatory cells (Razavi et al., 2011 ▶; Pirbalouti et al., 2009 ▶). Collagen is the main protein in the extracellular matrix and provides strength and integrity to the dermis (Pirbalouti 2011 ▶; Razavi et al., 2011▶; Pirbalouti et al., 2009 ▶). The result of macrophage histiocytic infiltration and neovascularization showed well-formation in the 10% *M. sylvestris* group, but not the SSD and other control groups. 

Our results also showed higher improvement of granulation tissue components such as collagen revenue in herbal treatment. Pirbalouti et al. reported that when using topical *M. sylvestris* in the treatment, collagen turnover significantly increased as compared to control groups. The findings of these two studies are similar (Pirbalouti and Koohpyeh 2011 ▶). 

Collagen increase is the main component of healing process which improves and supports extracellular tissue and wound healing. It can be concluded that this biological activity of the plant may help wound contraction and rate of healing burn wounds.

 Sayar et al. discussed that re-epithelialization occurred after 15 days with *Hypericum perforatum* (HP) treatment and 16.5 days with *Calendula* (as an herbal medicine) treatment (Sayar et al., 2014 ▶;), although re-epithelialization depends on thickness of the granular cell layer, epidermal thickness extent, maturation and organization of squamous cells, and migration of epithelial cells. Previous studies reported that re-epithelialization by some natural products in burn wound may be accelerated. Anti-inflammatory effect of some Malvacea and Boraginaceae species accelerated collagen fiber development and epithelium regeneration and improved epithelium thickness (Razavi et al., 2011 ▶; Mogosanu et al., 2013 ▶; Sayar et al., 2014▶;).

We found that re-epithelialization components in the *M. sylvestris* creams had a good epithelization on the 8th day after burn injury. These results showed no difference between 10% *M. sylvestris* and 5% *M. sylvestris* but the score in all control groups were lower as compared with herbal treatment groups. Moreover, we found that on 21^st^ day, the rats treated with 10% and 5% *M. sylvestris* creams had a mature new dermis formation. The control group exhibited a wide area of ulcerations, a mild degree of scar formation, and other new dermis components. In our study, the new dermis formation was significantly higher on day 21 in the *M. sylvestris* group compared to the others. Macrophage histiocytic infiltration with congestions in the dermis, indicated that the healing in 10% and 5% *M. sylvestris* was better than other control groups. This variable was equal between 10% *M. sylvestris* and 5% *M. sylvestris*. Therefore, this plant can be considered as a wound healing agent. The wound repair process treated with *M. sylvestris* group was better than the standard SSD group and others which involves steps including the degree of inflammation, mucopolysacharid deposition, fibroblastic proliferation, macrophage infiltration, degree of granulation tissue, and neovascularization. *M. sylvestris* cream increased neovascularization, short period epithelialization and improved healing of the infection. Collagen plays an important role in the wound healing and it is an important component of connective tissue, which affords a structural framework for the renewal tissue. Collagen is produced by fibroblasts and facilitates the wound in gaining good quality during wound healing. The process of wound healing is very complex and occurs through coagulation, inflammation, debridement, and re-epithelialization phases. The role of proliferation, migration, and discrimination of squamous epithelial cells of epidermis of the healing process is important. In the last stage of the healing process, collagen deposition and remodeling occurs intradermis (Fuller 2009 ▶; Mekonnen et al., 2013 ▶; Mustafa et al., 2013). In the present study, parameters of the granulation tissue and re-epithelialization on 8^th^ day post burn injury and parameters of new dermis on 21^st^ day were better in *M. sylvestris* treatment as compared to other treatment groups. In most of the cases, 10% *M. sylvestris* cream showed a better healing effect than other groups. The biological activity of this plant may be attributed to its antioxidants such as polyphenols, vitamin C, vitamin E, b-carotene, and other important phytochemical (Barros et al., 2010 ▶). In folk medicine, the medicinal application of the common mallow is to treat specific disorders such as digestive, respiratory, genitourinary, muscular, and skeletal system, as well as skin disorders and injuries. Moreover, it possesses anti-inflammatory properties. It is also used as bronchodilator, expectorant, antitussive, and anti-diarrheal. It is highly recommended for acne and skin care, for centuries by European, North African, and Asian people (Sayar et al., 2014 ▶). 

In our study, average of wound area, more than 90% percentage wound contraction, and wound healing time were 20 , 25, and 30-35 days for *M. sylvestris*, NS, BC, and SSD creams, respectively. This finding was similar to other reported studies (Veshkorova et al., 2010 ▶; Pirbalouti et al., 2009 ▶; Pirbalouti and Koohpyeh 2011 ▶). Nearly all histological data, including extent of granulation tissue (macrophage histocytic infiltration and neovascularization), re-epithelialization (epidermal thickness, thickness of granular cell layer, maturation organization of squamous cells, extent of keratin layer, and orthokeratin), and new dermis formation (degree of scar formation and collagenization organization) support the hypothesis that topical *M. sylvestris* cream is more efficient than standard common burn wound therapy in the treatment of second-degree burn wounds. The enhanced capacity for the histological promotion and acceleration of wound healing with the *M. sylvestris* could be explained by the anti-inflammatory, antibacterial, antioxidant properties, presence of mucilage, and high vitamin C content of this medicinal plant that is well documented in the previous studies (Barros et al., 2010 ▶; Pirbalouti and Koohpyeh 2011 ▶; Razavi al., 2011 ▶; Samavati and Manoochehrizade 2013 ▶; Pirbalouti et al., 2009 ▶).

The results of this study showed that *M. sylvestris* cream effectively improved various phases of the deep second burn wound and histology components of healing as compared to standard silver sulfadiazine and normal saline control groups. Therefore, this experimental study supports the recommendation of European, African, Asian, and Iranian traditional literatures about the use of this medicinal plant for wound healing. Topical administration of *M. sylvestris* cream resulted in faster healing burn wounds in vivo due to improvement in rates of wound contraction, facilitation of extent of granulation, reduction epithelialization time, new dermis formation, and prevention of infection burn wound parameters.
